# Development of GERAS DANcing for Cognition and Exercise (DANCE): a feasibility study

**DOI:** 10.1186/s40814-021-00956-3

**Published:** 2022-01-19

**Authors:** Patricia Hewston, Courtney Kennedy, George Ioannidis, Dafna Merom, Genevieve Hladysh, Sharon Marr, Justin Lee, Richard Sztramko, Laurel Trainor, Amanda Grenier, Matthew Harold Woolhouse, Christopher Patterson, Alexandra Papaioannou

**Affiliations:** 1 GERAS Centre for Aging Research, Hamilton Health Sciences, Hamilton, ON Canada; 2 Department of Medicine, McMaster University, Hamilton, ON Canada; 3 School of Science and Health , University of Western Sydney, Penrith, Australia; 4 YMCA Hamilton Burlington Brantford, Hamilton, ON Canada; 5 Department of Psychology, Neuroscience, & Behaviour, McMaster University, Hamilton, ON Canada; 6 Faculty of Social Work, University of Toronto, Toronto, ON Canada; 7 School of the Arts, McMaster University, Hamilton, ON Canada; 8 Department of Health Research and Methods, McMaster University, Hamilton, ON Canada

**Keywords:** Geriatrics, Cognition, Frailty, Rehabilitation, Dance

## Abstract

**Background:**

Dance is a mind-body activity of purposeful rhythmic movement to music. There is growing interest in using dance as a form of cognitive and physical rehabilitation. This manuscript describes the development of GERAS DANcing for Cognition and Exercise (DANCE) and evaluates its feasibility in older adults with cognitive and mobility impairments.

**Methods:**

The progressive dance curricula were delivered for 15 weeks (1-h class; twice weekly). Participants were eligible if they were community-dwelling older adults aged 60+ with early cognitive or mobility impairment able to follow three-step commands and move independently. Feasibility outcomes included recruitment/retention, adherence, participant satisfaction, safety, and adverse events.

**Results:**

Twenty-five older adults (mean (standard deviation [*SD*]) age = 77.55 (6.10) years, range 68–90 years) with early cognitive (Montreal Cognitive Assessment score (*SD*) = 21.77 (4.05)) and mobility (92% were pre-frail/frail as indicated on the Fried Frailty Phenotype) impairments were recruited from a geriatric out-patient clinic or within the community. A total of 20/25 (80%) participants completed the study. Average class attendance was 72%, and self-reported homework adherence “most-days / every day” was 89%. A stepwise progression in the dance curricula was observed with increases in motor complexity and balance demands, and 95% of participants rated the program as a “just-right” challenge. Ninety percent of participants rated GERAS DANCE as excellent, and 100% would recommend the program to a friend or family member. Over 50% of participants connected outside of class time for a self-initiated coffee club. Adverse events of falls and fractures were reported for 2 participants, which occurred at home unrelated to the dance intervention during the study period. Pre-determined thresholds for feasibility were met for all outcomes.

**Discussion:**

GERAS DANCE is a feasible and enjoyable program for older adults with early cognitive or mobility impairments. GERAS DANCE curriculum grading (duration; sequence; instructions) and motor complexity increases in agility, balance, and coordination appear appropriately tailored for this population. Future work will explore the feasibility of GERAS DANCE in new settings (i.e., virtually online, community centers, or retirement homes) and the mind-body-social benefits of dance.

**Supplementary Information:**

The online version contains supplementary material available at 10.1186/s40814-021-00956-3.

## Key messages regarding feasibility

- **What uncertainties existed regarding the feasibility?** GERAS DANCE is a new program designed with rehabilitation and geriatric medicine expertise for older adults with early cognitive or mobility impairments. Prior to completing this study, the feasibility and resources required to implement the GERAS DANCE program and the recruitment were uncertain.
- **What are the key feasibility findings?** Pre-determined thresholds for feasibility were met for all outcomes. GERAS DANCE curriculum grading (duration; sequence; instructions) and motor complexity increases in agility, balance, and coordination appear appropriately tailored for this population.
- **What are the implications of the feasibility findings for the design of the main study?** This study provides evidence that GERAS DANCE is a feasible and enjoyable program for older adults with early cognitive or mobility impairments. The next steps include testing the efficacy of GERAS DANCE in an RCT.

## Background

Both mild cognitive impairment (MCI) (16–20%) [1, 2] and frailty (10–15%) [3] are highly prevalent in community-dwelling older adults over the age of 60 and particularly for those over 85 years of age [4]. Cognitive and mobility impairments in older adults are associated with increased risk of disability, loss of independence [5], and impact on quality of life [6]. These impairments often co-exist, and their temporal relationship appears to be bi-directional [7]. Guidelines recommend that older adults with frailty exercise regularly as part of overall medical management [1, 3]. As outlined in the World Health Organization – Healthy Aging report [8], functional ability is the interaction between intrinsic capacity, environment, and task. Adherence and enjoyment increase when exercise is tailored to match functional abilities and delivered at an appropriate challenge and intensity level [9]. Given the demographic changes to world populations, there is an urgent need for action across multiple sectors for high-quality programs for older adults of all functional abilities.

Dance is a mind-body activity of purposeful rhythmic movement to music. Dance has been used as rehabilitation for people with Parkinson’s disease (PD) [10] and more recently for those with multiple sclerosis or stroke [11]. Dance for PD was designed to build upon strengths and anticipate perceptual-motor functional limitations of those with neurological conditions, creating a fun and safe environment to celebrate the artistry and movement [12]. Dance may also improve cognitive function [13], functional mobility and independence [14], muscular strength and endurance [15], and overall quality of life [16] in older adults. Furthermore, dance may be superior to traditional exercise programs given that it is an inherently complex sensorimotor activity that combines both physical and cognitive training as a single entity [17, 18] and added benefits of music [19]. Building upon these therapeutic benefits with rehabilitation and geriatric medicine expertise, we designed a program to bring the motivational enjoyment of dance aligned with dance for PD to community-dwelling older adults with early cognitive or mobility impairments. This manuscript describes the development of GERAS DANcing for Cognition and Exercise (DANCE) and evaluates its feasibility in older adults (aged 60+) with early cognitive or mobility impairments. Specifically, our objective was to determine the feasibility and resources required to implementing the GERAS DANCE and recruitment of older adults with early cognitive or mobility impairment using the 2010 Thabane et al. [20] and 2019 newly updated [21] guidelines and CONSORT checklist (Additional file 1).

## Methods

### Study design

We utilized a prospective cohort single-arm study, pre-post design, with a 15-week intervention phase.

### Participants and setting

Participants were recruited from a regional specialized geriatric clinic, community groups, and recreational centers, the Alzheimer’s Society utilizing flyers and social media. Referral sources were classified as clinical or community-based referrals. Inclusion criteria were (i) 60+ years of age and (ii) presence of early cognitive (Montreal Cognitive Assessment [MOCA] total score = 18–25) [22] or mobility impairments (e.g., difficulty to climb the stairs or walk around the block), (iii) ability to follow three-step commands, and (iv) ability to move independently with or without an assistive device. Exclusion criteria included (i) palliative/end-of-life care, (ii) unstable angina/heart failure, and (iii) travel plans that would result in missing more than 20% of the dance 15-week program. A research coordinator obtained informed consent from all participants in accordance with the Declaration of Helsinki and was approved by the Hamilton Integrated Research Ethics Board (HIREB #2557).

### Intervention

**Fig. 1:**
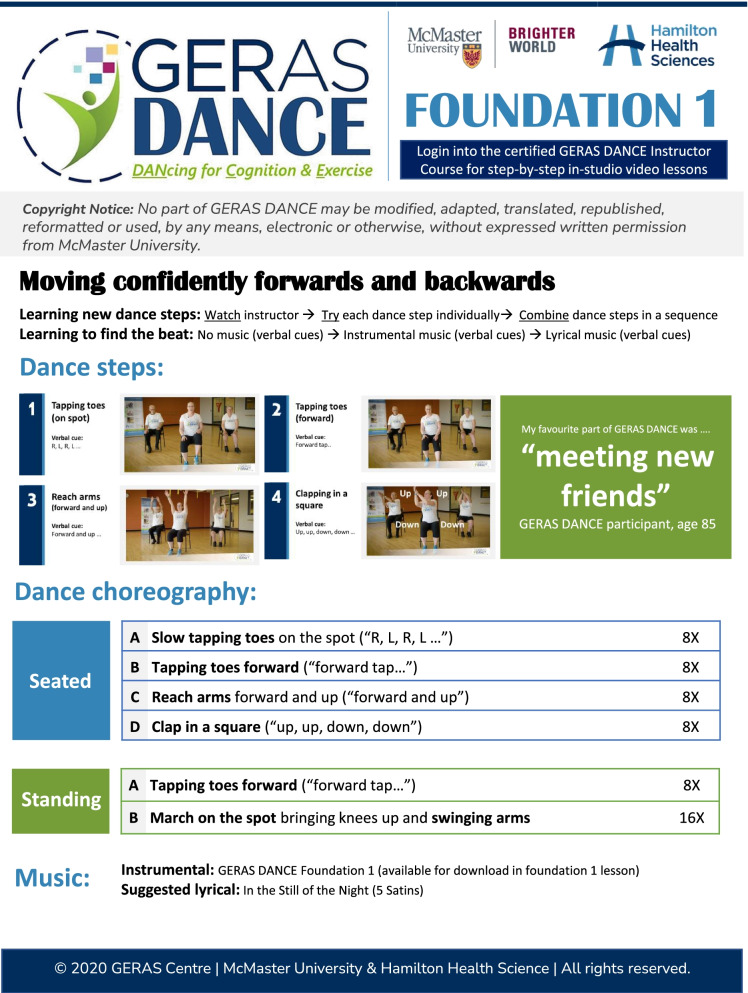
Sample GERAS DANCE curriculum

GERAS DANCE was designed with rehabilitation and geriatric medicine expertise as a 15-week mind-body program for older adults (aged 60+) with early cognitive or mobility impairments to make dance accessible for older adults with functional limitations (Fig. 1). GERAS DANCE was developed with an activity analysis [23], a systematic evaluation in the field of occupational therapy, to tailor the curriculum with appropriately matched grading (duration; sequence; instructions) and motor complexity increases in agility, balance, and coordination demands. It is a progressive, evidence-based curriculum with gradual increases in cognitive load and based on motor learning principles with a particular focus on the Agility, Balance, and Coordination (ABCs) of movement [24]. The ABCs of movement is essential to quickly and efficiently change the body position with balance and coordination [24, 25]. Dances were taught with a graded approach to learning each step (watch instructor; try each dance step individually; combine dance steps in a sequence) and find the beat (no music; instrumental music; lyrical music). The structured dance curriculum included both seated and standing dances (with and without holding onto the back of the chair), gradually increasing the time spent in standing with increased endurance. This graded approach to teaching new dance steps maximized the likelihood of success by emphasizing having fun while learning something new (rather than the perfection of movement). Repetition was used to build confidence in movement patterns, and verbal cues were used to help execute dances.

**Fig. 2:**
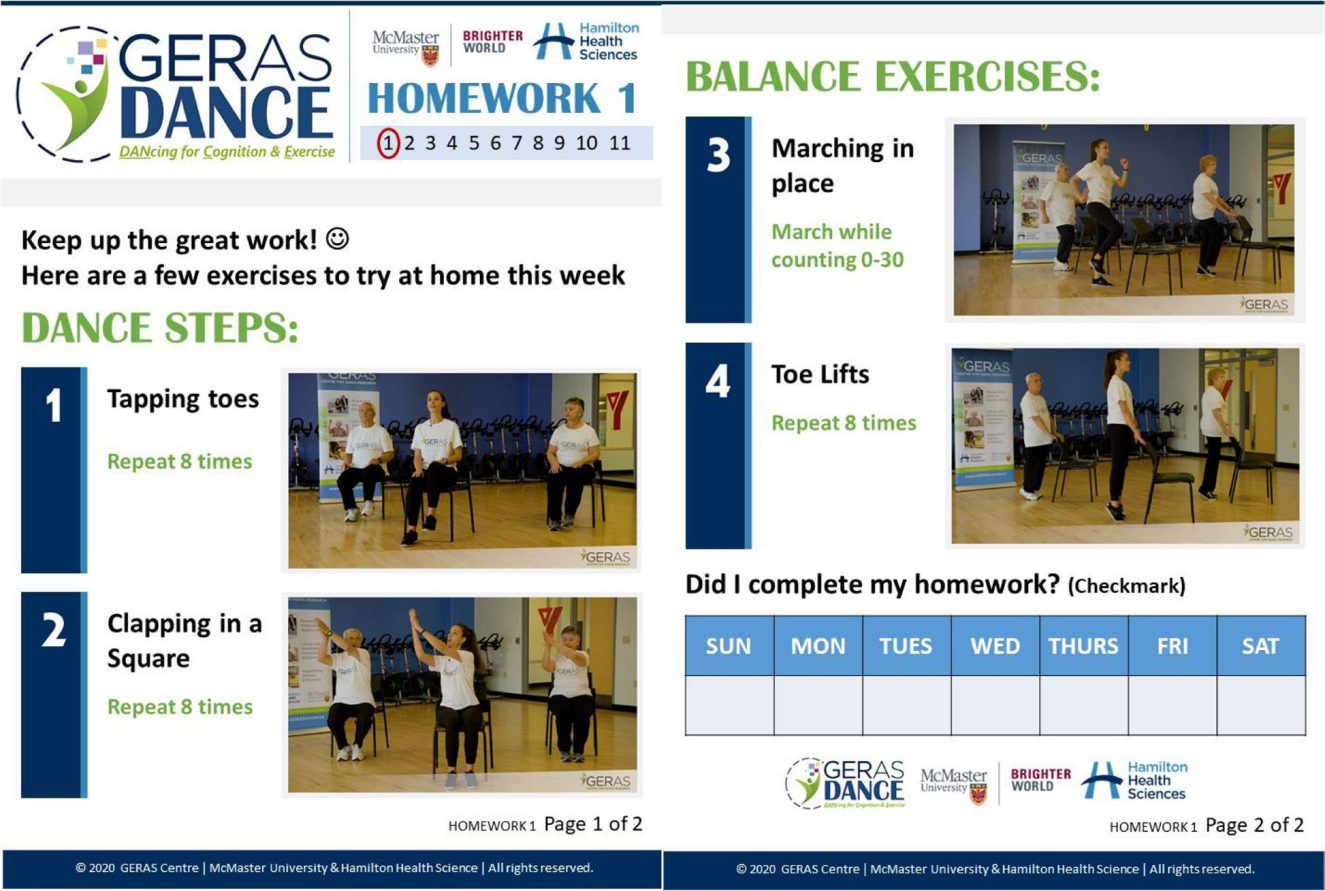
Sample GERAS DANCE homework

GERAS DANCE was delivered as a new program at the Young Men’s Christian Association (YMCA) Hamilton Burlington Brantford as a part of the LiveWell series, which includes tailored rehabilitation programs for people with cancer [26] or heart disease [27]. GERAS DANCE was taught by a dance instructor who adhered to a standardized curriculum documented in a manual. GERAS DANCE involved 15 weeks of in-person classes 2× weekly (1 h each) and homework (10 min daily). Each in-person class followed the same sequence of activities (introduction/socialization [10 min]; warm-up [5 min]; structured dance curriculum [30 min]; cool-down [5 min]; review of weekly homework [10 min]). The curriculum schedule included five foundations and seven routines. GERAS DANCE foundations emphasized learning the ABCs of movement and increasing participants’ confidence to move their bodies forward and backward and side-to-side with improved speed and rhythmicity. GERAS DANCE routines combined the foundational skills into full choreographed dances to music from the 1950s and 1960s (e.g., rock and roll, jazz, salsa, cha cha cha, rhythm soul, swing, and disco). The last 2 weeks of the curriculum was used to repeat the favorite class dances. Homework sheets for non-class days (target 10 min daily) outlined simple instructions and pictures to reinforce/practice movements learned and achieve 180 min per week, to reduce falls in older adults (Fig. 2) [28]. Instructor and end-user feedback was utilized to adapt GERAS DANCE programming to match the functional abilities of the cohort. A Template for Intervention Description and Replication (TIDieR) checklist is available in Additional file 2 and preview at www.gerascentre.ca/geras-dance.

### Training, equipment, and facilities

The instructor and volunteers received training about working with older adults with early cognitive or mobility impairments including safety and communication strategies. Before each GERAS DANCE class, volunteers ensured that the studio was clear of tripping hazards (e.g., gym mats) and that sturdy chairs without armrests were set up in a semi-circle formation. A sound system with adjustable volume and a headset microphone was required for the instructor adjusted for older adults with hearing impairment. Using Whitehead et al. [29] and Thabane et al. [20] feasibility guidelines, our sample size was pragmatic and based on class enrollment capacity (25 participants maximum per class) aligned with infrastructure and safety guidelines at the YMCA. This sample was adequate to determine the feasibility and resources required to implement the GERAS DANCE program and the recruitment process in planning for a larger trial.

### Participant demographics

Demographic information was collected at baseline, including age, sex, education level, living arrangement, and fall history in the past year. Cognition was evaluated with the Montreal Cognitive Assessment (MoCA) [30]. Total MOCA scores range from 0 to 30, and 18–25 points adjusted for educational level are suggestive of mild cognitive impairment [22, 30]. The Fried Frailty Phenotype evaluated frailty status based on unintentional weight loss (greater than 10 lbs in the past year), low endurance and energy (self-reported exhaustion), weakness (low grip strength), slow walking speed, and physical activity (self-reported activity level). An overall score of 0 means that the individual is not frail; a score of 1 or 2 indicates that the individual is pre-frail; a score of 3–5 indicates frailty. Physical function was evaluated with the Short Physical Performance Battery (SPPB), and a score of < 9 points is indicative of poor physical performance [31]. Cognitive, frailty, and physical function were assessed by a research assistant not involved in the study intervention at the GERAS Centre for Aging Research.

### Feasibility outcomes

#### Recruitment and retention

The enrollment-to-screening ratio was calculated as the total number enrolled as a proportion of the number of individuals identified to meet the inclusion criteria. The recruitment period was calculated as the time between recruitment start and completion. The eligibility criteria will be considered sufficient if the recruitment rate is > 10%. The retention rate was calculated as the number of individuals who remained in the study out of the total number of participants recruited at the baseline assessment. The retention rate will be considered sufficient > 75% based on recommendations in Thabane et al. [20].

#### Intervention adherence, safety, and adverse events

Adherence to the GERAS DANCE classes was recorded by the instructor using an attendance log and calculated as the percentage of classes attended (*n*/30). Adherence to the homework was recorded using a self-reported questionnaire. Exercise adherence in older adults in the literature ranged from 65 to 86%, with lower adherence rates observed in those with lower cognitive and physical health [32]. Therefore, in this study, adherence greater than 65% of the GERAS DANCE classes and homework will be considered sufficient due to the increased number of barriers to exercise adherence in our cohort (e.g., poor cognitive/physical health, fatigue, low self-efficacy, lack of transportation, reliance on a care partner) [33, 34]. An instructor log was used to record any modifications required to match functional abilities. Adverse events (e.g., falls, fatigue, etc.) were self-reported and recorded by the dance instructor.

#### Participant satisfaction

At the last GERAS DANCE class, participants were asked to complete a feedback questionnaire designed to assess the elements of the following item of GERAS DANCE: (1) overall program rating: a 5-point Likert scale from terrible, poor, fair, good, or excellent; (2) elements of the program enjoyed (yes/no); (3) perceived level of challenge: indicating if the level of the classes was too easy, just right, or too hard; (4) program recommendation: would recommend GERAS DANCE to a friend or family member (yes, no, maybe); (5) reason for recommendation (fitness; social; learn something new, fun, other); and (6) if the program led to social connections outside the class (yes/no).

#### Statistical methods

Descriptive statistics were used to describe the participant characteristics and feedback including means, standard deviations, 95% confidence intervals, and ranges. All descriptive statistics were calculated using SPSS v25.

## Results

### Participant demographics

Of the participants who completed the study (*n* = 20), 70% were female and had a mean age of 77.55 years (*SD* = 6.10, range 68–89; Table 1). Falls were common, with 10/20 (50%) of participants indicated that they had experienced a fall in the past year, and 5/20 (25%) had experienced two or more falls in the past year. Assessments indicated 70% had evidence of cognitive impairment (MOCA < 26), and 50% had mobility limitations (SPPB < 9). Four of the 20 participants who completed the study were two married couples. Five participants did not complete the study; the reasons for withdrawals were fall and fracture (*n* = 2; fell down the stairs due to stroke and passed away a month later, and by tripping over a slipper at home), not interested in dance (*n* = 1), lack of transportation (*n* = 1), and unknown reason (*n* = 1).

**Table 1:** Baseline descriptives

	Completed (***n*** = 20)	Did not complete (***n*** = 5)
Age years (*SD*)	77.55 [95% *CI* 74.80, 80.31]	81.80 [95% *CI* 76.29, 87.31]
Sex (% female)	14/20 (70%)	4/5 (80%)
Living arrangement (lives alone)	5/20 (25%)	2/5 (40%)
*Education* more than high school (% of participants)	7/20 (35%)	3/5 (60%)
*Falls*: no falls in the past year (% of participants)	5/20 (25%)	3/5 (60%)
*Falls*: > 1 fall in the past year (% of participants)	10/20 (50%)	1/5 (20%)
*Falls*: > 2 falls in the past year (% of participants)	5/20 (25%)	1/5 (20%)
*Frailty*: Fried frailty (pre-frail or frail)	19/20 (95%)	4/5 (80%)
*Cognition*: MOCA total score (*SD*)	22.47 [95% *CI* 20.49, 24.45]	19.40 [95% *CI* 15.74, 23.06]
*Physical function*: SPPB total score (*SD*)	8.25 [95% *CI* 7.31, 9.19]	9.60 [95% *CI* 7.72, 11.48]

Baseline descriptives of older adults who completed the study compared to those who did not as indicated by adherence to the GERAS DANCE classes greater than 70%

*ICON-FES* 10-item Iconographical Falls Efficacy Scale, *MOCA* Montreal Cognitive Assessment, Fried Frailty Phenotype, *SPPB* Short Physical Performance Battery, *CI* confidence interval

### Recruitment and retention

**Fig. 3:**
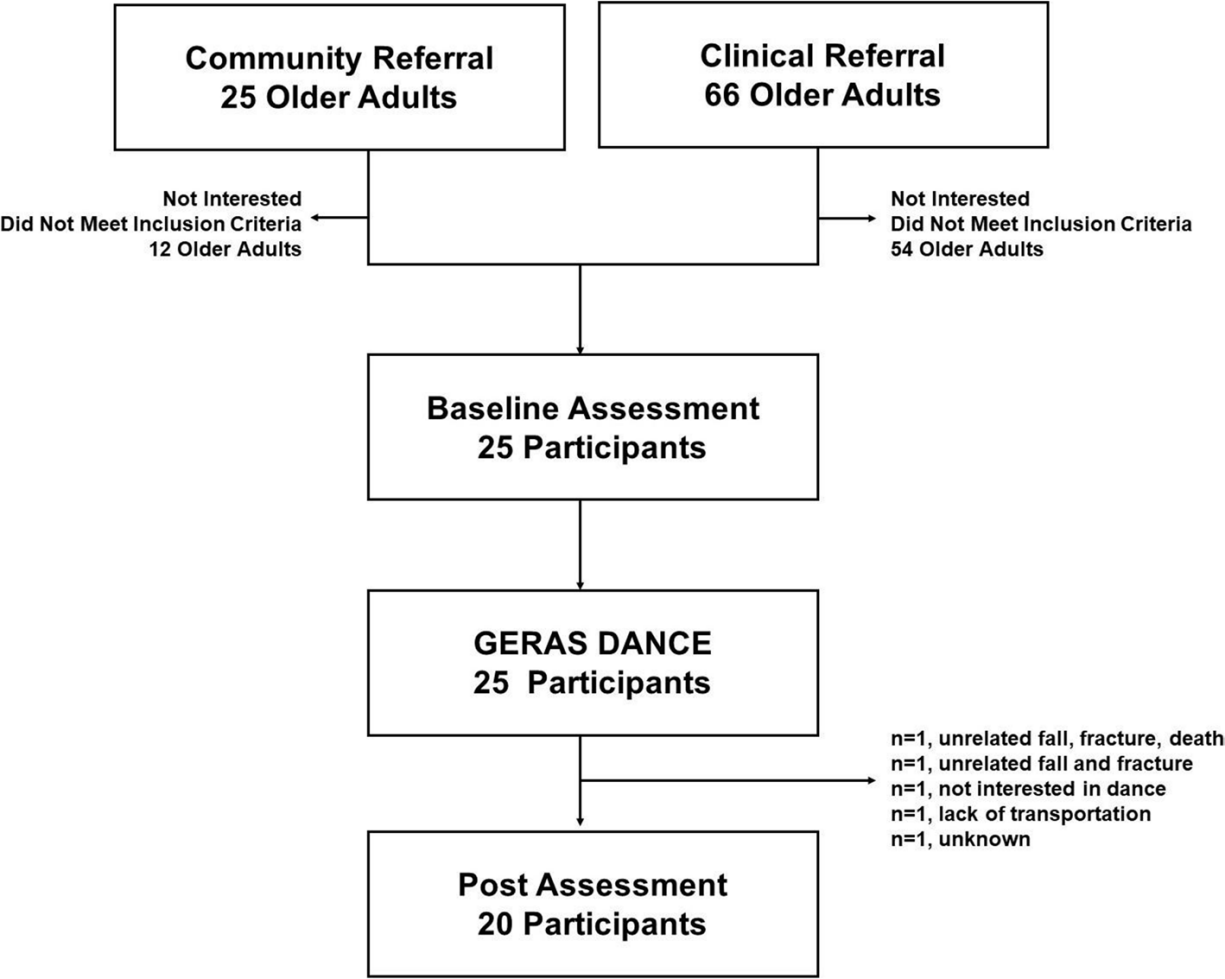
Participant flow

Figure 3 outlines enrollment and retention. Referral sources were 48% (12/25) clinical and 52% (13/25) community-based referrals. For the clinical referrals, 66 geriatric out-patients were referred and met the inclusion criteria and of those, 12 (18.2%) were enrolled in the study. The enrollment-to-screening ratio was calculated as 2:11. For community-based self-referrals, of the 20 phone calls received, 13 (65%) were enrolled in the study. The recruitment period occurred over 3 months, and the participant retention rate was 80%.

### Intervention adherence, safety, and adverse events

The average class attendance of the study cohort was 72%, and self-reported homework adherence “most-days / every day” was 89%. A stepwise progression in the dance curricula was observed with increases in motor complexity and balance demands (1 class to learn each foundation and 2+ classes to learn the routines). Instructor notes indicated the music from the 1950s and 1960s was enjoyed by the attendees. All participants were able to complete seated and standing dances. However, some participants who felt tired used the option to remain seated during some of the standing dance segments. The default dance step was marching in the spot to the beat if participants needed to rest or felt the steps were too complicated. Instructor notes indicated for ease of scheduling, 12 weeks was preferred over 15-week classes. One caregiver initially attended with their husband to ensure they were comfortable participating in the first three classes. One fall happened during the dance class due to an untied shoelace, but no injuries were sustained. Participants did not engage in other new interventions beyond GERAS DANCE during the study period.

### Participant satisfaction

Table 2 outlines responses on the feedback questionnaire. Ninety percent of participants rated GERAS DANCE as excellent. All participants (100%) enjoyed the dancing standing up, music, instructor, social time, class time (1–2 pm), and class length (1 h). Only 70% of participants enjoyed dancing while sitting in a chair. Ninety-five percent of participants rated the perceived level of challenge as “just right,” and 100% would recommend the program to a friend or family member. Reasons for giving a recommendation included exercise (89%), social (74%), learning something new (68%), and fun (68%). Other written responses for recommendation were the quality of the instructor, interacting with people, watching others on how they do it, encouraging me, and laughing with friends. Over 50% of participants connected outside of class time for a self-initiated coffee club and were individuals who did not previously know each other before the GERAS DANCE class.

**Table 2:** Participant feedback [*N* = 20]

**Overall program rating (% of participants who rated the program as …)**	
- Terrible	*0%*
- Poor	*0%*
- Fair	*5%*
- Good	*5%*
- Excellent	*90%*
**Enjoyment of program components (% of participants who …)**	
- Enjoyed the warm-up	95%
- Enjoyed dancing in a chair	70%
- Enjoyed the dancing standing up	100%
- Enjoyed the music	100%
- Enjoyed the instructor	100%
- Enjoyed the social time	100%
- Enjoyed the class location (local YMCA)	95%
- Enjoyed the class time (1–2 pm)	100%
- Enjoyed the class length (1 h)	100%
**Overall perceived level of challenge (% of participants who …)**	
- Too easy	*5%*
- Just right	*95%*
- Too hard	*0%*
**Program recommendation (% of participants who said …)**	
- Yes, would recommend to a friend or family member	*100%*
**Reason for recommendation (% of participants who would recommend for …)**^**a**^	
- Exercise/fitness	*89%*
- Social/meet new people	*74%*
- To learn something new	*68%*
- For fun	*79%*
- Other responses included instructor, interacting with people, watching others on how they do it, others encouraging me, and laughing with friends	*N/A*
**Social connections (% of participants)**	
- Yes, I connected outside of classes with other participants	*53%*

^a^Note this was a supplemental open ended in the feedback questionnaire

## Discussion

This manuscript provides evidence that GERAS DANCE is a feasible and enjoyable program for older adults with cognitive and mobility impairments (aged 68–90 years) with high participant adherence and satisfaction. GERAS DANCE curriculum grading (duration; sequence; instructions) and motor complexity increases in agility, balance, and coordination appear appropriately tailored for this population. The program requires a large space, sturdy chairs, and a sound system with a microphone.

Given the importance of a clinical trial recruitment phase, it is critical to understand the feasibility, timelines, and best strategies to enroll participants [21]. The study recruitment period occurred over 3 months to register 25 older adults with early cognitive or mobility limitations. For future trials, a stronger focus on community-based recruitment may help to reduce research staff time and improve the recruitment rate. Clinical referrals from family physicians may be more appropriate for the prevention and rehabilitation of cognitive impairment and frailty. The retention rate of participants was 80%, which is aligned with a recent systematic review on exercise adherence in older adults ranging from 65 to 86% [32**]**.

GERAS DANCE programming aligns with recent evidence suggesting dance programs be organized to run twice per week, 1 to 2 h in length, for 3 months in duration to optimize social, physical, and cognitive benefits in neurological populations [11, 35]. GERAS DANCE involves multicomponent exercise with both in-studio and at-home components to achieve the recommended 180 min per week to reduce falls in older adults [28]. In the in-studio classes, each dance included seated and standing components. Only 70% of participants enjoyed the seated dances, whereas 100% enjoyed the standing dances. However, as Gregor et al. [29] stated, we agree that an initial sense of physical safety while seated may have increased confidence to test intrapersonal boundaries (fear of falling) to explore new movement patterns while standing confidently. GERAS DANCE uses a graded approach to teaching further dance steps and an emphasis on having fun while learning something new which may have increased overall enjoyment and confidence by the time the standing dances were performed [36]. Instructors provided the option to remain seated while dancing when fatigued, check for tripping hazards, and remind participants to ensure their shoelaces were tied before standing up. The GERAS DANCE homework component was well-received, with 89% of participants completing the homework most days or every day *to* reinforce/practice movements learned. Participants were able to participate in the classes without the support of a caregiver. One caregiver initially attended with their husband to ensure they were comfortable participating in the classes; however, after three classes, the caregiver started to use this as respite time to go to the library.

Ninety-five percent of participants rated GERAS DANCE as a just-right challenge, and 100% would recommend the program to a friend or family member. Compared to the Baycrest Canada National School of Ballet Sharing Dance Seniors program [37], where participants co-construct and collaboratively animated the narrative of the dances, GERAS DANCE has a progressive and structured curriculum aligned with dance movement therapy. Recommendations for GERAS DANCE extended beyond the exercise component itself (e.g., social, learning something new, laughing with friends). The instructor notes indicated that the attendees enjoyed music from the 1950s and 1960s, which aligns with the participant’s musical “reminiscence bumps,” in which music is associated with events in late adolescence and early adulthood [38]. The power of dance in fostering new social connections and a sense of community was further demonstrated as 50% of participants connected for a self-initiated coffee club during and beyond the study duration. It is recommended to teach the holistic mind-body-social benefits of dance when training new instructors to understand the program benefits beyond the exercise itself. Lastly, GERAS DANCE duration was recommended to be reduced from 15 weeks as implemented in this study to 12 weeks to better align with the YMCA programming schedule with other programs which are 12 weeks in length.

### Limitations

The GERAS DANCE program has the potential to be implemented as community-based rehabilitation. This research study was supported by grant funding, which allowed participants to attend without any financial cost. If participants did not receive free memberships, the enrollment could have been lower than reported here. It is possible that dancing may not be an appealing exercise to men as a higher percentage of females were involved in the study. Future work should include a cost analysis and potential funding mechanisms to provide the dance program as a community-based program as a part of overall rehabilitation.

## Conclusion

GERAS DANCE is a promising therapeutic intervention and enjoyable for older adults with cognitive and mobility impairments. Future work should explore the feasibility of GERAS DANCE in new settings—i.e., virtually online, community centers, or retirement homes. GERAS DANCE efficacy will be studied in a randomized controlled trial to examine the mind-body-social benefits of dance.

## Supplementary Information

**Additional file 1.** CONSORT 2010 checklist of information to include when reporting a pilot or feasibility trial.

**Additional file 2.** TIDieR (Template for Intervention Description and Replication) Checklist.

## Data Availability

The datasets generated during and analyzed during the current study are not publicly available (individual privacy of the participants could be compromised) but are available from the corresponding author on reasonable request.

## Abbreviations

ABCs: *A*gility, *B*alance, and *C*oordination
GERAS DANCE: GERAS DANcing for Cognition and Exercise
MCI: Mild cognitive impairment
MOCA: Montreal Cognitive Assessment
PD: Parkinson’s disease
SD: Standard deviation
SPPB: Short Physical Performance Battery
YMCA: Young Men’s Christian Association
